# How funding agencies can support research use in healthcare: an online province-wide survey to determine knowledge translation training needs

**DOI:** 10.1186/1748-5908-9-71

**Published:** 2014-06-06

**Authors:** Bev J Holmes, Megan Schellenberg, Kara Schell, Gayle Scarrow

**Affiliations:** 1 Michael Smith Foundation for Health Research, Vancouver, BC, Canada; 2 Mental Health Commission of Canada, Ottawa, Ontario, Canada

**Keywords:** Knowledge translation, Evidence-based decision making, Health research capacity building, Health research funder, Evidence use

## Abstract

**Background:**

Health research funding agencies are increasingly promoting evidence use in health practice and policy. Building on work suggesting how agencies can support such knowledge translation (KT), this paper discusses an online survey to assess KT training needs of researchers and research users as part of a Canadian provincial capacity-building effort.

**Methods:**

The survey comprised 24 multiple choice and open-ended questions including demographics, interest in learning KT skills, likelihood of participating in training, and barriers and facilitators to doing KT at work. More than 1,200 people completed the survey. The high number of responses is attributed to an engagement strategy involving partner organizations (health authorities, research institutes, universities) in survey development and distribution. SPSS was used to analyze quantitative results according to respondents’ primary role, geographic region, and work setting. Qualitative results were analyzed in NVivo.

**Results:**

Over 85 percent of respondents are interested in learning more about the top KT skills identified. Research producers have higher interest in disseminating research results; research users are more interested in the application of research results. About one-half of respondents require beginner-level training in KT skills; one-quarter need advanced training. Time and cost constraints are the biggest barriers to participating in KT training. More than one-half of respondents have no financial support for travel and almost one-half lack support for registration fees. Time is the biggest challenge to integrating KT into work.

**Conclusions:**

Online surveys are useful for determining knowledge translation training needs of researchers, research users and ultimately organizations. In this case, findings suggest the importance of considering all aspects of KT in training opportunities, while taking into account different stakeholder interests. Funders can play a role in developing new training opportunities as part of a broad effort, with partners, to build capacity for the use of health research evidence. Survey results would ideally be complemented with an objective needs assessment based on core competencies, and should be acted on in a way that acknowledges the complexity of knowledge translation in healthcare, existing training activities, and the expertise stakeholders already have but may not refer to as knowledge translation.

## Background

Health research funding agencies in Canada are increasingly focused on knowledge translation (KT), in part to demonstrate accountability for spending public dollars, but also recognizing they are well placed to facilitate the movement of evidence into practice and policy.

Because these agencies are funded by provincial governments, there is increased pressure to target resources towards resolving health system issues. The gap between the evidence generated and that which is applied in healthcare is becoming too large to ignore. KT, with its focus on helping researchers and research users (practitioners and decision makers) work together to create and apply knowledge, is seen as a way to reduce this gap. KT is variously described, but a definition increasingly used in Canada is attributed to the Canadian Institutes of Health Research (CIHR): a dynamic and iterative process that includes synthesis, dissemination, exchange and ethically-sound application of knowledge to improve the health of Canadians, provide more effective health services and products and strengthen the healthcare system [1].

KT has the potential to decrease adverse effects on patients and use taxpayers’ dollars more efficiently [2-4]. A growing literature is exploring the barriers to the dissemination and use of evidence by both researchers and those who use research. These barriers include lack of access to information, lack of knowledge and skills, inadequate infrastructure to support evidence-informed decision making, lack of strong leadership, lack of incentives, and intervention characteristics. This literature also suggests which mechanisms might overcome specific barriers in a range of settings, although the evidence is far from clear and this work is nascent [2,5-11].

**Figure 1:**
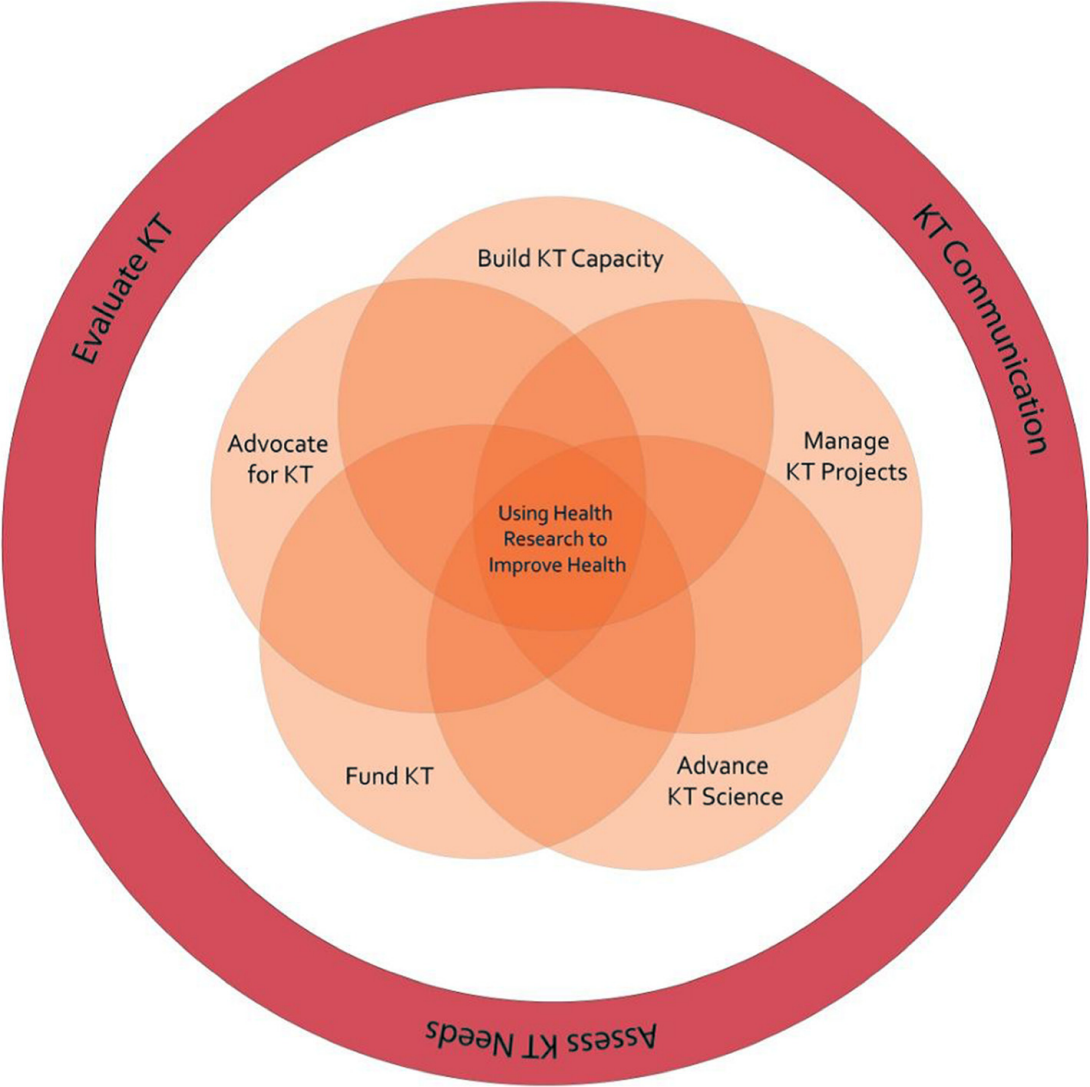
KT roles for health research funding agencies.

In an earlier article, [12] we discussed the work of one funding agency in British Columbia (BC), Canada, to support the use of health research evidence. Figure 1 presents our model comprising five key functional areas in which funding agencies can work: advancing KT science; building KT capacity; managing KT projects; funding KT activities; and advocating for KT. We suggest that together, these functions create an environment conducive to KT. The model acknowledges the complexity of using research evidence in healthcare, including how evidence and knowledge are conceptualized by diverse stakeholders [13-15].

This article describes a provincial needs assessment we undertook towards strengthening one of the five functional areas—building KT capacity, acknowledged as critical to improving the quality of healthcare [16]. We sought to identify KT training needs of both researchers and research users (healthcare practitioners and decision makers), given that both these groups, if supported appropriately, can play a key role in the dissemination and implementation of knowledge. Wilson *et al.* (2010) suggest more could be done—by funders, in particular—to require and support researchers to engage in KT in consistent ways. As for research users, from policy makers to decision makers to clinicians, evidence points to some of the barriers that prevent their engagement in KT (5,7,10). Although not all these barriers are easily overcome, learning opportunities are one mechanism that could be useful [17,18]. Our needs assessment was one step towards developing these opportunities [17].

The term capacity building is widely used—particularly in health promotion and international development [19]—and its definitions are many. For our definition, we adapted one component of a framework developed through our agency’s Health Authority Capacity Building (HACB) program [20]. Although the framework encompasses research capacity building broadly, our interest was in the component ‘developing skills through providing training,’ which includes activities aimed at building appropriate knowledge, skills, and confidence in individuals through training opportunities and educational resources. This component is similar to the top two levels of Potter and Brough’s [19] four-tier hierarchy of capacity building needs: structures, systems, and roles; staff and facilities; skills; and tools. The model’s four tiers are interconnected in a logical hierarchy that depicts how each tier is dependent on, and builds on, lower tiers and their effectiveness towards capacity building. For example, for tools (the top of the hierarchy) to be effective they require that components of lower tiers (skills; staff and facilities; and structures, systems and roles) be appropriate and effective; however, components of lower tiers do not require higher tiers to exist and/or be effective in order to be effective capacity builders themselves. We appreciate the importance of the lower two tiers, and the warning of the authors that skills training alone will never sustain capacity; accordingly, training is only one part of the KT capacity building under consideration by our agency. We agree with Davis and Davis [17], however, that educational modalities may play a crucial role in predisposing to change more broadly. This change may be within individuals, but also organizationally and even beyond, helping to build momentum for other key areas of KT support such as leadership and technical infrastructure.

## Methods

We developed a self-administered online survey as the most appropriate mechanism to understand KT training and resource needs in BC. Online surveys can elicit a high volume of feedback in a short amount of time from people who live in geographically separated areas. They can also be targeted to specific groups of people who share a common interest [21]. Challenges to using an online survey include the inability to control who responds, and the exclusion of those without access to the Internet [22] or who are unable to use online survey tools such as SurveyMonkey due to their organizations’ privacy policies. Such challenges could potentially lead to sampling issues. However, the survey was only the first step of a multi-phased project with opportunities for further needs assessment, so these challenges were not a concern. In our view, the potential negative aspects of online surveys (inability to estimate population parameters, perception as junk mail, skewed attributes of Internet population, privacy issues and low response rate) were outweighed by Evans’ [22] list of ‘best uses’ for Internet surveys, including where wide geographic coverage is sought; a large sample is desired; and there is access to a good sample list.

Our intended audience was people who produce and/or use health research evidence as part of their work and—due to the complex nature of the subject—who are aware of the field of KT. We acknowledge there are many producers and users of health research evidence who are not aware of KT but who would appreciate access to training. An online survey is not the best way to determine the needs of this group because of potential unfamiliarity with the terminology and concepts used by those in the KT field. Other approaches are clearly needed to tap into their expertise and ideas about building KT capacity in BC.

Survey objectives were to determine KT training needs of communities that produce and use health research evidence, identify interest in KT training opportunities, and ask about barriers to KT training as well as KT practice. A review of both the Tri Council Policy Statement on *Ethical Conduct for Research Involving Humans* [Article 2.5] [23] and completion of the University of British Columbia’s checklist for studies requiring ethical review [24] indicated ethics approval was not required for our needs assessment.

The survey questions were developed based on the KT expertise and experience of the authors who are members of our organization’s KT and evaluation staff. Survey questions, specific KT skills and barriers were brainstormed as they related to KT in general as well as the four components as defined by CIHR [1]: dissemination, synthesis, exchange, and application (Table 1). We considered design factors such as quality of questions, survey format and the way questions are presented [22]. Before releasing the survey broadly, we pilot tested it with a group of KT colleagues across Canada. The feedback was extensive, with some suggestions easy to incorporate (*e.g.*, edits for clarity), and others requiring a great deal of thought (*e.g.*, challenges about how to conceptualize KT).

**Table 1:** KT Definitions

**Dissemination**	Identifying the appropriate audience and tailoring the message and medium to the audience. Dissemination activities can include such things as summaries for/briefings to stakeholders, educational sessions, creation of tools and media engagement.
**Synthesis**	The contextualization and integration of research findings of individual research studies within the larger body of knowledge on the topic. A synthesis must be reproducible and transparent in its methods, using quantitative and/or qualitative methods. It could take the form of a systematic review, follow the methods developed by the Cochrane Collaboration, result from a consensus conference or expert panel or synthesize qualitative or quantitative results. Realist syntheses, narrative syntheses, meta-analyses, meta-syntheses and practice guidelines are all forms of synthesis.
**Exchange**	Interactions between evidence users and researchers at any or all stages of the research process. The Canadian Health Services Research Foundation (CHSRF) says that effective knowledge exchange involves interaction between knowledge users and researchers and results in mutual learning through the process of planning, producing, disseminating, and applying existing or new research in decision-making.
**Application**	The iterative process by which new or existing health research evidence is put into practice. Application can refer to both the integration of evidence into existing programs, policies or practices, or the development of new evidence-informed programs, policies, practices, products and services.

The final survey included 24 multiple choice and open-ended questions [see Additional file 1]. Informed consent was not sought from respondents given that the survey was anonymous (*i.e.*, no identifying information was collected) and its main purpose was a needs assessment. Demographic questions included primary professional role, geographic region (by the province’s five regional health authority areas), and workplace setting.

The first section of the survey focused on respondents’ interest in various KT skills in the categories of general KT, dissemination, synthesis, exchange, and application. Working definitions were provided for knowledge translation and each of the four components to establish common and clear understanding among respondents (Table 1). Respondents rated the importance of these components to their work on a scale of four from not important to very important. Respondents were also asked to record whether they were interested or not interested in learning any of 28 KT skills listed, and if they were interested, to self-assess what level of training they believe they needed (beginner, intermediate or advanced). A second section asked about KT support. Respondents were asked to pick their top two preferred learning formats from a list of options and to record the likelihood of their engaging in specific activities on a six-point scale (from very unlikely to very likely). In this section, they were also asked how much support they receive in their organization to participate in KT training (from no support to full support with regard to time, encouragement, registration fees and travel costs). They were also asked to check all that apply from a list of factors that would prevent participation. Finally, respondents rated seven barriers to doing KT in their work on a scale from one (not a barrier) to five (a major barrier). An open-ended question invited comments about what would support respondents to practice KT in their work.

We chose to use a communications strategy as a practical approach to recruitment based on our knowledge of the BC KT landscape and players. Dissemination of the survey began with targeted emails tailored to key stakeholder groups. We identified well-known and respected ‘KT champions’ within each BC geographic health region who were asked to distribute the link to the survey under their own signatures within their organizations. We also identified a broad list of researchers funded by our agency, as well as other research community stakeholders, who were asked to complete the survey and forward it to others; and we emailed ‘key influencers’ (leaders of health research-related organizations) who would support the survey. Reminder emails were sent two weeks later. The survey was live for three weeks and was promoted through social media, our agency’s e- newsletter, and our website. Some of our partners also promoted the survey through their organizational channels.

Of the 1,206 responses received, 1,071 met the criteria for inclusion in our analysis (*i.e.*, more than the demographics section was filled out, and respondent located in BC). We used SPSS to analyze quantitative results. Overall frequencies provided us with initial findings, and based on demographic information, we further broke down data to understand different patterns by primary role, geographic region, and setting. We analyzed qualitative results, including ‘other’ responses, in NVivo using a thematic analysis that categorized comment concepts (for example, ‘time,’ as one of the barriers to doing KT at work).

To understand differences between those who more generally produce research and those who use it, we regrouped professional roles by categories of research producers (researchers, clinician-scientists, and research trainees) and research users (healthcare providers and administrators, public servants, and knowledge brokers). We considered knowledge brokers (intermediaries between producers and users of research) a unique group because of their hands-on role in KT, and therefore in some instances separated them. For a more nuanced understanding of the differences between respondents’ primary professional roles, we also compared seven unique groups—researchers, clinician-scientists, research trainees, healthcare providers, healthcare administrators, public servants, and knowledge brokers.

## Results

### Demographics

**Figure 2:**
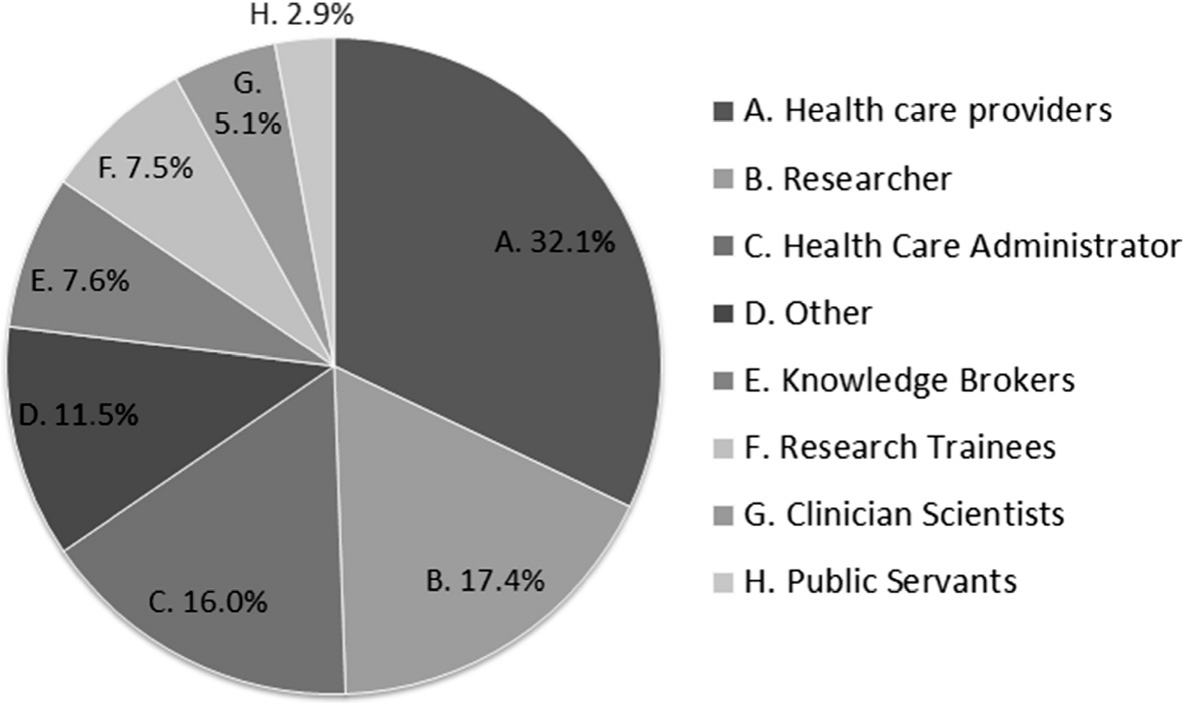
Respondents by primary professional role.

Forty-eight percent of respondents were research users (healthcare providers, 32 percent; administrators, 16 percent) primarily working in health authorities, and 30 percent were research producers (researchers, 17 percent; research trainees, eight percent; clinician-scientists, five percent) primarily from universities or research institutes. Of researchers and trainees, 29 percent do biomedical research, 25 percent do health services research, 24 percent do population health research, and 16 percent do clinical research; the remainder are in other or multiple areas. Eight percent of respondents overall were knowledge brokers, three percent were public servants and 12 percent identified as ‘other’ including clinical educators, research administrators and other support functions (see Figure 2). Participants were from geographic regions across the province, with 50 percent based in two of the province’s major urban regions (Vancouver and the Fraser region), 21 percent from the north of the province, 17 percent on Vancouver Island, and 12 percent in the province’s Interior region.

### Importance of KT and interest in learning more

All four aspects of KT—dissemination, synthesis, exchange, and application—were rated as important to respondents’ work, with dissemination and application the highest importance (3.36 out of four) followed by exchange (3.27) and synthesis (2.94). Research producers consider dissemination the most important aspect of KT, while research users consider application to be of most importance. Regional results about the importance of KT to respondents’ work likely reflect differences in their roles. For example, dissemination is rated most important on Vancouver Island and in Vancouver, where there are more respondents who are researchers and trainees. In the other regions, where respondents were predominantly healthcare providers and administrators, application was rated most important.

Across all the KT skills, 80 percent of respondents are interested in learning more. Of the top 10 skills identified (see Additional file 2), all are of interest to over 85 percent of respondents. The highest overall interest is skill-building related to knowledge exchange (82 percent), followed by skills related to dissemination (82 percent), application (81 percent), general KT (79 percent) and synthesis (72 percent). Percentages were calculated based on the average figure across all sub-items in that group. Forty-seven percent of respondents who indicated interest in learning more said they require beginner-level training. On average, 23 percent of respondents require training in KT skills at the advanced level.

Comparing the KT interests of the various roles, knowledge brokers indicated the most interest in learning more about KT across all skills (more than 90 percent interest in approximately two-thirds of the skills). There is generally high interest (over 80 percent) from both research producers and users in nine specific KT skills, most of which coincide with the top 10 skills overall. Research producers have higher levels of interest in general KT and dissemination skills, while research users have higher levels of interest in exchange and application skills. On average, 63 percent of healthcare providers require beginner-level training in KT skills, compared to the overall average of 47 percent. Even in the area of application, which is rated of most importance to research users, the percentage of healthcare providers who need beginner-level training is higher than all other roles.

### KT learning preferences and barriers to training

The most preferred learning format is small group sessions, chosen by 72 percent of respondents. Self-guided study is the second most preferred format (47 percent), followed by teleconferences and webinars (34 percent), large group sessions (29 percent), and one-on-one formats (12 percent). Compared to the overall average, more healthcare administrators (42 percent), public servants (37 percent) and healthcare providers (37 percent) prefer to learn with teleconferences and webinars, while fewer clinician-scientists (18 percent), research trainees (26 percent), researchers (28 percent) and knowledge brokers (34 percent) indicated these two formats were a preference.

With regard to likelihood of engaging in various KT learning activities on a scale of six (from very unlikely to very likely), the highest percentage of respondents said they would be somewhat to very likely to access online resources (86 percent), attend a workshop (84 percent) and take free web-based KT training with local and international mentors and peers (84 percent). To a lesser extent, respondents indicated they would likely take free web-based KT training for a certificate (72 percent), seek KT advice (67 percent), work with a KT mentor (68 percent), join a KT community of practice (63 percent), and apply for KT funding (62 percent). Respondents’ roles impact their likelihood of engaging in most activities, with knowledge brokers and research trainees more likely to engage than the overall average. Researchers and clinician-scientists are less likely to engage in the various KT activities, with results below the overall average in all areas except applying for KT funding.

Only five percent of respondents indicated that nothing would prevent them from participating in training. Relating to specific barriers, 34 percent indicated that lack of commitment from an employer would prevent them from participating. All other barriers are comparable: travel costs (64 percent), multi-day time commitment (59 percent), location (59 percent) and registration fees (57 percent). Primary professional roles impact the way individuals responded to some of the barriers. Higher percentages of healthcare providers (53 percent) and public servants (48 percent) indicated that lack of commitment from an employer would prevent their participation; more clinician-scientists (72 percent) identified a multi-day commitment as a barrier; and a higher percentage of research trainees (76 percent) said registration fees would prevent them from participating. In addition, more respondents from rural and remote settings said location and a multi-day commitment are barriers than respondents from urban centres. With regard to cost, most respondents said they would attend a training workshop only if the cost was less than $200 Canadian. More people are also interested in workshop training over a shorter period of time: five percent would not attend a one-day training workshop, while 29 percent would not attend a workshop over three days.

Overall, competing priorities is the biggest barrier to doing KT in work, with 75 percent of respondents choosing four or five on the scale. Of the remaining barriers, funding for KT activities (68 percent) and access to KT resources (52 percent) are the other top issues. Primary roles impact how individuals perceive the levels of barriers in all areas except time/competing priorities. Healthcare providers and administrators generally rate barriers higher than the overall results, with healthcare providers consistently above. Researchers and public servants generally rate barriers below the overall results. An open-ended question asked about facilitators to doing KT in the workplace, which 18 percent of respondents answered. Nearly one-half of the 18 percent identified resources of varying types that would increase their capacity to do KT, including additional people, dedicated staff, expertise through mentors, librarians, and KT units. In other cases, respondents said that tools and KT materials would support their KT activities, as would funding for training, people, and resources. Reflecting the biggest barrier identified, approximately one-quarter of respondents’ comments reiterated the need for more time. About one-quarter of the comments focused on organizational culture as a facilitator to doing KT.

## Discussion

In a recent study of Canadian healthcare organizations, Ellen *et al.*[18] describe four key categories of KT supports for evidence-informed decision-making: roles that promote research use; ties to researchers outside the organization; technical infrastructure; and training programs to enhance staff capacity building. The survey described in this paper aims to increase understanding of how best to implement the fourth category, with the important caveat that training is ideally only one component of a broader strategy to increase the individual, organizational and in this case, provincial use of research evidence.

Our expectation of response numbers was modest, given survey fatigue, busy schedules, potential confusion over KT terminology or frustration with its perceived jargon, the survey length, and what seems to be a growing discomfort in the Canadian healthcare and academic sectors with data stored outside Canada (we used SurveyMonkey, which is based in the U.S.). Despite these concerns, the number of responses surpassed our expectations. We attribute the rate to the KT champions who distributed the link to the survey under their own signatures within their organizations. The keys to their doing so were our existing partnerships aimed at increasing evidence use in BC, our engagement with them throughout the development of the survey, our offer to provide regional results for their own use, and our intent to support them in delivering KT training programs and services based on the findings. While results include a good balance between respondents who are research producers and research users across the province, understanding of KT training needs of groups that were not as well represented in our survey (not-for-profit, government and private sectors) will be important.

That most respondents consider KT important to their work and are interested in learning more about it is perhaps not surprising, given that people who complete surveys tend to have a high interest in the topic [21,22]. This interest is consistent with our experience to date in offering general KT skills workshops, which have been oversubscribed. The survey described in this paper was designed to move beyond general interest to specific training needs as well as other aspects important to training.

Four out of five respondents are interested in each KT skill listed, suggesting that demand would be high for any of the topics offered via training. Research producers are most interested in learning more about dissemination and general KT skills; research users are most interested in learning more about application and exchange skills. While these findings can be acted on, it will be important to bear in mind the subjectivity of this needs assessment and the importance of an objective component [17], for example based on core competencies [25]. Of note is that respondents rate the importance of KT to their work as high, yet the highest demand for training is at the beginner level. This may suggest that people do not feel knowledgeable enough to perform the KT tasks necessary for their roles. The fact that the survey relied on respondents to interpret ‘beginner, intermediate, and advanced’ training levels makes this finding difficult to interpret; see more on this finding in Strengths and Limitations, below. Also of note is that knowledge exchange is rated third in ‘importance to respondents’ work’ of the four components of KT (dissemination, synthesis, exchange, and application), yet had the highest overall interest in terms of skill-building. We are unclear as to the reason for this discrepancy. It may relate to the order and/or specificity of the survey questions themselves. That is, the question ‘How important to your work is knowledge exchange’ was asked first and included a general definition and description of ‘exchange’. Immediately following this question respondents’ were asked to indicate their interest in learning more about six very specific training topics related to exchange (see Additional file 1). The specificity of the topics themselves may have served to spark interest in respondents in a way that a general definition and description of exchange did not.

Survey findings suggest that training formats should be flexible, easily accessed, and cost-effective. There is most interest in small group sessions as a learning format, and more likelihood that people will attend a KT workshop over most other activities. While results confirm the value of workshops in the province, it is also apparent that cost constraints and time commitments are the biggest barriers to participating in them. Offering a range of options—in terms of formats as well as fees—will increase the likelihood that more people can participate. It will be important to explore creative training models in order to move beyond more traditional didactic training where participants with similar backgrounds ‘learn and leave,’ with little opportunity to apply what they have discovered or talk about their experiences as they attempt to incorporate what they have learned in practice. For example, training that offers a mix of workshop-based and practice-based components, and that provides ongoing mentorship and specific problem-based learning, could be explored. A mix of participants in terms of professions could help build understanding of cultural and language differences, encourage integrated and end-of-grant KT activities and partnerships, and address a perceived barrier noted in our survey by healthcare providers and administrators—and to a lesser extent researchers—for opportunities to interact. Before designing training opportunities, further exploration of the evidence that shows what is most effective for whom will be necessary. For example, small group learning, distance education and communities of practice show promise for healthcare practitioners [17], while tools provided by funding agencies are recommended as a strategy for supporting researchers’ learning [11]. The preferences of policymakers, and best practices in training for this group, seems to be less clear. Indeed, existing literature on training tends to explore opinions, ideas, and likelihood of participating, rather than effectiveness of training formats and topics themselves. There is a huge opportunity in launching a KT training program to explore what works best for whom, as well as immediate, medium and longer term outcomes.

Given the high interest in KT training, finding enough trainers will be a challenge. Possibilities include encouragement and incentives for local KT leaders to share their knowledge and experience, and exploring the addition of train-the-trainer components into existing and new training initiatives. Building on existing training initiatives, and working across the province on a shared overall training program with flexibility for local adaptations, has the potential to maximize expertise and also resources as well as enable the setting of common objectives and measurement indicators.

Finally, as indicated earlier, while KT skills training is a key component of organizations’ support for evidence based decision making [18], it alone is not sufficient to increase the use of health research evidence. Some respondents in their comments drew a connection between organizational culture and time: without increased awareness and understanding of KT from leadership and staff, commitment to making KT a priority from management and others, and supportive structures that allow or require KT in the workflow, time will continue to be an issue. Just as one training intervention is not enough to result in a meaningful change in performance [3], building KT skills is only one component of developing a more supportive environment for KT. While funding agencies can address some organizational barriers (*e.g.*, access to resources, opportunities for interactions between researchers and users of evidence, KT funding), results suggest the importance of working with partner organizations to address context-specific barriers to practicing KT and, importantly, to evaluate how specific mechanisms promote research engagement by organizations [26].

### Strengths and Limitations

Strengths of the survey described in this paper include the large response rate, broad focus, and ability to determine differences among professional groups [27]. An additional strength is the positioning of the survey as one component that will help build health research capacity provincially, which will force us to look at training in context of other support necessary. While we hope these strengths will offset the limitations of this survey, these must still be acknowledged.

Despite the high number of responses, because of the nature of surveys—primarily quantitative, with little opportunity to understand the subtleties of responses—there is a need for caution in interpreting the findings so as not to overlook their complexity. For example, we were interested in the seeming disconnect between the fact that respondents consider KT important to their work, but that the highest demand for training is at the beginner level. We noted that healthcare practitioners were consistently more likely to report needing beginner level training than the average. One might conclude that responsibility for KT is included in many job descriptions, but people do not know enough about how to do it. However, this conclusion would probably be false. Healthcare practitioners are expert at incorporating many types of evidence and knowledge, both tacit and explicit, into their work (Vicky Ward, personal communications, 2012). Ward questions whether KT has been professionalized to such an extent that people think there it is a ‘right’ way to do it that they need to be taught. We agree with Ward on the importance of legitimizing the ways in which people are already using evidence in practice and policy, and supporting them to learn more from each other, ideally in the way they are already practicing.

Another complexity results from using an online survey to find out about a topic as complicated as knowledge translation. Despite our efforts to use jargon-free language, we do not know that people understood the questions exactly as we meant them to be understood. Twelve people commented that the language in the survey was inaccessible. Admittedly, 12 out of nearly 1,100 people is not a high percentage, but these were people who chose to comment (*i.e.*, respondents were not asked about the language level of the survey), and presumably these were also people who are interested in and therefore know something about KT. It is conceivable that even our efforts at clarity resulted in some misunderstandings. It is certainly the case that these efforts painted a less-than-ideal picture of KT from our perspective. For example, we were not entirely happy with dividing KT into four areas—dissemination, exchange, synthesis and application—because in practice these are artificial distinctions and can lead to the perception that KT is a linear process starting with knowledge generation and ending with its application [21]. However, we did need to ask about interest in specific skills as there are important distinctions among them in terms of learning; therefore we compromised our position.

Two more factors suggest caution in interpreting the findings. Although a lot of information was generated by many people, there are a) many things we did not hear, and b) stakeholders from whom we heard little. On the first point, the survey format and length did not allow for gathering information about, for example, how many courses a year might people take, or whether they would prefer a basic understanding of KT over in-depth training on one aspect, or how we might build on their existing knowledge and resources they have used. On the second point, despite a good response rate from many stakeholder groups, others were under-represented, for example, policy makers in government. Another group that was under-represented were healthcare providers whose work involves ‘translating knowledge’ but who may not be familiar with KT terminology and may have little interest in learning it or no patience for its perceived jargon. We were urged by a few respondents to find ways other than the survey to explore the needs and existing expertise of these stakeholders. For these and other reasons, we would not consider our results to be generalizable beyond those who answered the survey. However, we do suggest that the findings are robust enough to proceed with the development of training opportunities in partnership with healthcare and research organizations around the province.

## Conclusions

There are challenges to meeting the increasing political and societal expectations that research findings should generate a return on investments in the form of improved health and social and economic outcomes. Among them, research takes time to realize results, and implementation of those results into the complex system that is healthcare is not a linear process involving discrete, predictable and entirely manageable stages [28,29].

The field of KT promises to address these challenges in a number of ways, one of which is through training to support the use of evidence for researchers and research users. This paper outlines the efforts of one funding agency and its partners to address the acknowledged shortage of people skilled in KT practice and science [25] by building capacity through training and resources. Given the high response to our survey and the interest in KT across Canada and nationally, we believe this is an opportune time for health research funders and other organizations involved in KT to determine how best to work in partnership to support evidence use in health practice and policy making.

## Competing interests

The authors declare they have no competing interests.

## Authors’ contributions

BH contributed to the design of the survey and wrote the paper. MS contributed to the design of the survey and co-wrote the paper. KS designed the survey, conducted the analysis and contributed to revisions of the paper. GS contributed to the design and analysis of the survey and provided revisions for the paper. All authors read and approved the final manuscript.

## Supplementary Material

Additional file 1 BC Knowledge translation needs assessment. Click here for file

Additional file 2 Top ten KT skills. Click here for file
