# Supplementary material for: How funding agencies can support research use in healthcare: an online province-wide survey to determine knowledge translation training needs
Source: Implement Sci. 2014 Jun 6;9:71. doi: 10.1186/1748-5908-9-71 (PMC4060070; doi:10.1186/1748-5908-9-71)
Supplement: Additional file 1 — BC Knowledge translation needs assessment. [file 1748-5908-9-71-S1.pdf]

BC Knowledge Translation Needs Assessment

INTRODUCTION

As BC's provincial health research support agency, MSFHR is committed to supporting the use of health research evidence to improve practice and policy (knowledge translation, or KT).

Please join your colleagues in identifying KT training and resource needs across BC. We're looking for input from both "producers" and "users" of health research evidence, recognizing that many of you play both roles.

The results of this survey will help us and our partners develop KT training and resources of most interest to respondents.

Please note:

- We see knowledge translation as a complex process whose components are not in practice easily - or ideally - separated. ***However***, the survey asks specific questions about evidence dissemination, synthesis, exchange and application to understand needs in these specific areas as well as for KT overall.
- We also recognize that many types of evidence must be drawn on to improve health care practice and policy. ***The focus of this particular survey*** is health research evidence (or results of health research), recognizing its effective use relies on other types of evidence in context.

Thanks in advance for your input. We'll post a summary of the results on our web page this spring.

Use the navigation buttons in the survey. You can leave and return to your responses at a later time using the same computer and survey link. It should take approximately 15 minutes to complete. Please note: There is no identifying information collected in this survey. For more information, visit our website.

ABOUT YOU

BC Knowledge Translation Needs Assessment

1. What is your primary professional role?

- Researcher
- Clinician-scientist
- Research trainee (graduate or post-graduate)
- Health care provider
- Health care administrator
- Public servant (i.e. working in government)
- Knowledge broker (i.e. intermediary between researchers and evidence users)
- Other (please specify)

2. What is your primary work environment?

- University/college
- Research institute (within a health authority or university)
- Not-for-profit organization
- Health authority (including hospital and community)
- Government
- Private sector
- Other (please specify)

3. Where is your workplace located?

- A (Northern Health geographic region)
- B (Vancouver Coastal Health geographic region)
- C (Fraser Health geographic region)
- D (Vancouver Island Health Authority geographic region)
- E (Interior Health geographic region)
- Outside BC

(BC Stats, 2002)

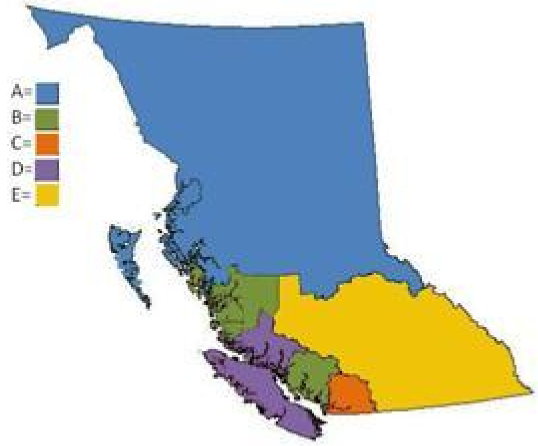

4. In what setting is your workplace located?

- ◯ Urban
- ◯ Rural or remote

5. What type of research most closely aligns with your work?

- ◯ Not applicable
- ◯ Don't know
- ◯ Biomedical research
- ◯ Clinical research
- ◯ Health services research
- ◯ Population health research
- ◯ Other type of research (please specify)

The survey is in two parts. Part I asks about your interest in learning more about KT broadly as well as specific aspects of KT. Part II asks about training experiences, preferences, and barriers to KT.

For the purposes of this survey we use the definition of knowledge translation as developed by Canadian Institutes of Health Research:

> *A dynamic and iterative process that includes synthesis, dissemination, exchange and ethically-sound application of knowledge to improve the health of Canadians, provide more effective health services and products, and strengthen the health care system. This process takes place within a complex system of interactions between researchers and knowledge users which may vary in intensity, complexity and level of engagement depending on the nature of the research and the findings as well as the needs of the particular*

knowledge user.

Other terms commonly used are knowledge transfer, knowledge exchange, research utilization, and dissemination and implementation (US).

PART I. KNOWLEDGE TRANSLATION

6. Indicate if you're interested in learning more about the following, and if so the level of training you need.

Select all that apply.

	Interested (beginner)	Interested (intermediate)	Interested (advanced)	Not interested
Developing a KT plan	◯	◯	◯	◯
Implementing a KT plan	◯	◯	◯	◯
Evaluating a KT plan	◯	◯	◯	◯
KT models and theories	◯	◯	◯	◯
KT research (also known as dissemination and implementation research)	◯	◯	◯	◯
Teaching KT	◯	◯	◯	◯

Other topics of interest (please specify)

DISSEMINATION

Identifying the appropriate audience and tailoring the message and medium to the audience. Dissemination activities can include such things as summaries for/briefings to stakeholders, educational sessions, creation of tools and media engagement.

7. How important to your work is dissemination, as defined above?

- ◯ Not important
- ◯ Somewhat important
- ◯ Moderately important
- ◯ Very important

8. Indicate if you're interested in learning more about the following, and if so the level of training you need.

Select all that apply.

	Interested (beginner)	Interested (intermediate)	Interested (advanced)	Not interested
Developing a dissemination plan	◯	◯	◯	◯
Implementing a dissemination plan	◯	◯	◯	◯
Evaluating a dissemination plan	◯	◯	◯	◯
Developing key messages	◯	◯	◯	◯
Communicating using plain language (written and verbal)	◯	◯	◯	◯
Targeting communication to specific audiences	◯	◯	◯	◯
Social marketing (i.e. the systematic application of marketing to achieve specific behavioral goals for a social good)	◯	◯	◯	◯
Working with the media	◯	◯	◯	◯

Other topics of interest (please specify)

SYNTHESIS

The contextualization and integration of research findings of individual research studies within the larger body of knowledge on the topic. A synthesis must be reproducible and transparent in its methods, using quantitative and/or qualitative methods. It could take the form of a systematic review, follow the methods developed by the Cochrane Collaboration, result from a consensus conference or expert panel or synthesize qualitative or quantitative results. Realist syntheses, narrative syntheses, meta-analyses, meta-syntheses and practice guidelines are all forms of synthesis.

9. How important to your work is synthesis, as defined above?

- ◯ Not important
- ◯ Somewhat important
- ◯ Moderately important
- ◯ Very important

10. Indicate if you're interested in learning more about the following, and if so the level of training you need.

Select all that apply.

	Interested (beginner)	Interested (intermediate)	Interested (advanced)	Not interested
Conducting evidence syntheses	◯	◯	◯	◯
Communicating evidence syntheses (e.g. summaries, overviews, policy briefs)	◯	◯	◯	◯
Finding and appraising evidence syntheses	◯	◯	◯	◯

Other topics of interest (please specify)

EXCHANGE

Interactions between the evidence user and the researcher at any or all stages of the research process. The Canadian Health Services Research Foundation (CHSRF) says that effective knowledge exchange involves interaction between knowledge users and researchers and results in mutual learning through the process of planning, producing, disseminating, and applying existing or new research in decision-making.

11. How important to your work is knowledge exchange, as defined above?

◯ Not important

◯ Somewhat important

◯ Moderately important

◯ Very important

12. Indicate if you're interested in learning more about the following, and if so the level of training you need.

Select all that apply.

	Interested (beginner)	Interested (intermediate)	Interested (advanced)	Not interested
Working with decision makers	◯	◯	◯	◯
Working with researchers	◯	◯	◯	◯
Working with industry	◯	◯	◯	◯
How decisions are made in health care environments	◯	◯	◯	◯
How decisions are made in government environments	◯	◯	◯	◯
Using social media or web-based tools for knowledge exchange (e.g. web-based forums, wikis, social networking sites)	◯	◯	◯	◯

Other topics of interest (please specify)

APPLICATION

The iterative process by which new or existing health research evidence is put into practice. Application can refer to both the integration of evidence into existing programs, policies or practices, or the development of new evidence-informed programs, policies, practices, products and services.

13. How important to your work is application, as defined above?

- Not important
- Somewhat important
- Moderately important
- Very important

14. Indicate if you're interested in learning more about the following, and if so the level of training you need.

Select all that apply.

	Interested (beginner)	Interested (intermediate)	Interested (advanced)	Not interested
Developing evidence-informed practices and programs				
Implementing evidence-informed practices and programs				
Sustaining evidence-informed practices and programs				
Evaluating the implementation of evidence-informed practices and programs				
KT related to the commercialization of products or services				

Other topics of interest (please specify)

PART II. KT SUPPORT

KT support includes learning resources, advice, training activities, funding, etc.

15. Please rate your level of agreement with the following.

In my work environment, the KT support available is generally:

	Strongly disagree	Disagree	Somewhat disagree	Somewhat agree	Agree	Strongly agree	Don't know
Accessible	◯	◯	◯	◯	◯	◯	◯
Affordable	◯	◯	◯	◯	◯	◯	◯
Relevant	◯	◯	◯	◯	◯	◯	◯
Interesting	◯	◯	◯	◯	◯	◯	◯
Of high quality	◯	◯	◯	◯	◯	◯	◯
Of a sufficient amount	◯	◯	◯	◯	◯	◯	◯

16. List up to five of the most useful KT resources and activities you have used or heard of.

17. List up to three KT skills trainers (based in Western Canada) you would recommend to lead a KT workshop.

KT SUPPORT: PREFERENCES

18. How do you prefer to learn about a topic?

Select up to two.

- ☐ Self-guided study (e.g. reading, video, etc.)
- ☐ Large group sessions (e.g. seminars, conferences, community of practice)
- ☐ Small group sessions (e.g. workshops, seminars)
- ☐ One-on-one
- ☐ Teleconferences and webinars
- ☐ Other (please specify)

19. What is the likelihood that you would engage in the following?

	Very unlikely	Unlikely	Somewhat unlikely	Somewhat likely	Likely	Very likely	Don't know
Attend a KT workshop	◯	◯	◯	◯	◯	◯	◯
Take brief, free web-based KT training with local and international mentors and peers	◯	◯	◯	◯	◯	◯	◯
Study for and obtain free web-based KT training with local and international mentors and peers that would lead to a certificate	◯	◯	◯	◯	◯	◯	◯
Access online KT resources (e.g. KT models, tools and best practice information)	◯	◯	◯	◯	◯	◯	◯
Join a KT community of practice/network	◯	◯	◯	◯	◯	◯	◯
Work with a KT mentor	◯	◯	◯	◯	◯	◯	◯
Seek KT advice (e.g. call a help desk)	◯	◯	◯	◯	◯	◯	◯
Apply for KT funding	◯	◯	◯	◯	◯	◯	◯

Other (please specify)

20. How much of the following do you receive at your place of work to participate in a KT training workshop?

	No support	Some support	Full support	Not sure
Encouragement	◯	◯	◯	◯
Time	◯	◯	◯	◯
Registration fees	◯	◯	◯	◯
Travel costs	◯	◯	◯	◯

21. What would prevent your participation in a KT training workshop?

Select all that apply.

- ☐ Multi-day time commitment
- ☐ Registration fees
- ☐ Location
- ☐ Travel costs
- ☐ Lack of commitment from employer
- ☐ Nothing would prevent me from participating.

Other (please specify)

22. How much would you/your employer be willing to pay in registration fees so you could attend a:

	$0 - Only if it was free	Up to $200	Up to $600	Up to $1000	Up to $1600	I would not attend
1-day training workshop?	◯	◯	◯	◯	◯	◯
2-day training workshop?	◯	◯	◯	◯	◯	◯
3-day training workshop?	◯	◯	◯	◯	◯	◯
5-day KT professional certificate course?	◯	◯	◯	◯	◯	◯

BARRIERS

23. On a scale from one to five, how much of a barrier is each of the following to doing KT in your work?

	1 = not a barrier	2	3	4	5 = a major barrier	Not applicable
KT skills of staff	◯	◯	◯	◯	◯	◯
Time/competing priorities	◯	◯	◯	◯	◯	◯
Formal recognition from employer	◯	◯	◯	◯	◯	◯
Organizational culture	◯	◯	◯	◯	◯	◯
Opportunities to interact with researchers and evidence users	◯	◯	◯	◯	◯	◯
Funding for KT activities	◯	◯	◯	◯	◯	◯
Access to KT resources	◯	◯	◯	◯	◯	◯

Other (please specify)

24. What else do you require in your work environment to better support KT in your work?

THANK YOU

Do you have any additional comments regarding your needs or preferences related to KT?

BC Knowledge Translation Needs Assessment

Thank you for your input!

Please click the button below to submit your survey.

For more information about this survey or MSFHR's knowledge translation activities, visit our website or contact Gayle Scarrow, Knowledge Translation Manager.
