# Supplementary material for: How funding agencies can support research use in healthcare: an online province-wide survey to determine knowledge translation training needs
Source: Implement Sci. 2014 Jun 6;9:71. doi: 10.1186/1748-5908-9-71 (PMC4060070; doi:10.1186/1748-5908-9-71)
Supplement: Additional file 2 — Top ten KT skills. [file 1748-5908-9-71-S2.pdf]

Additional file 2: Top ten KT skills

Area	KT Skills	Top ten ranking	% Interested in learning more	% Research Producer[a] interest	% Research User[b] interest
General KT	Developing a KT plan		84.4		
	Implementing a KT plan	10	85.2		
	Evaluating a KT plan		83.0		
	KT models and theories		81.5		
	KT research		79.0		
	Teaching KT		57.8		
Dissemination	Developing a dissemination plan		83.1		
	Implementing a dissemination plan		84.7		
	Evaluating a dissemination plan		83.1		
	Developing key messages	8	85.4		
	Communicating using plain language	4	87.4		
	Targeting communication to specific audiences	3	87.8		
	Social marketing		75.2		
	Working with the media		67.8		
Synthesis	Conducting evidence syntheses		69.9		
	Communicating evidence syntheses		75.4		
	Finding and appraising evidence syntheses		70.7		
Exchange	Working with decision-makers	2	88.7		
	Working with researchers		81.5		
	Working with industry		68.0		
	How decisions are made in health care environments	1	89.2		
	How decisions are made in government environments		83.9		
	Using social media or web-based tools for knowledge exchange		82.7		
Application	Developing evidence-informed practices and programs	5	86.7		
	Implementing evidence-informed practices and programs	6	86.7		
	Sustaining evidence-informed practices and programs	7	86.4		
	Evaluating the implementation of evidence-informed practices and programs	9	85.3		

	KT related to the commercialization of products or services		57.5		

[a] Includes researchers, clinician-scientists and research trainees

[b] Includes health care providers and administrators, and public servants. For analysis on levels of interest, knowledge brokers were considered separately because of consistently high interest.

(dark purple)	80% interest and over
(light purple)	70 – 79% interest
(light blue)	Below 70%
(light green)	Varying results
